# Impact of Heat and Drought Stress on Grasspea and Its Wild Relatives

**DOI:** 10.3390/plants12193501

**Published:** 2023-10-08

**Authors:** Khawla Aloui, Hasnae Choukri, Noureddine El Haddad, Priyanka Gupta, Keltoum El Bouhmadi, Peter M. F. Emmrich, Akanksha Singh, Anne Edwards, Fouad Maalouf, Outmane Bouhlal, Jasmine Staples, Shiv Kumar

**Affiliations:** 1 International Center for Agricultural Research in the Dry Areas (ICARDA), Rabat 10112, Morocco; hasnae_choukri@um5.ac.ma (H.C.); noureddine_elhaddad@um5.ac.ma (N.E.H.); o.bouhlal@cgiar.org (O.B.); 2 Laboratory of Ecology and Environment, Ben M’Sick Faculty of Sciences, University Hassan II, Casablanca 20800, Morocco; keltoum.elbouhmadi@gmail.com; 3 Laboratoire de Biotechnologie et de Physiologie Végétales, Centre de Recherche BioBio, Faculté des Sciences, Mohammed V University Rabat, Rabat 10112, Morocco; 4 Département de phytologie, Institut de Biologie Intégrative et des Systèmes Pavillons Charles-Eugène Marchant, Université Laval, Québec, QC G1V 4G2, Canada; prgup1@ulaval.ca; 5 John Innes Centre, Norwich Research Park, Norwich NR4 7UH, UK; peter.emmrich@jic.ac.uk (P.M.F.E.); anne.edwards@jic.ac.uk (A.E.); jasmine.staples@jic.ac.uk (J.S.); 6 International Center for Agricultural Research in the Dry Areas (ICARDA), New Delhi 110012, India; a_singh1388@yahoo.in; 7 International Center for Agricultural Research in the Dry Areas (ICARDA), Beirut 1108 2010, Lebanon; f.maalouf@cgiar.org

**Keywords:** grasspea, ODAP content, heat stress, drought stress, grain yield, crude protein

## Abstract

Grasspea (*Lathyrus sativus* L.) is recognized as a highly drought-tolerant legume. However, excessive consumption of its seeds and green tissues causes neurolathyrism, a condition characterized by an irreversible paralysis of the legs induced by a neurotoxin amino acid called β-N-oxalyl-L-α, β- diaminopropionic acid (β-ODAP). The present study investigated the effects of heat, and combined heat + drought during the reproductive phase on physiological and phenological parameters, yield-related factors, ODAP content, and seed protein of 24 genotypes representing 11 *Lathyrus* species under controlled conditions. Analysis of variance revealed a highly significant effect (*p* < 0.001) of stress treatments and genotypes for all the traits. In general, heat stress individually or in combination with drought expedited phenology, reduced relative leaf water content, stimulated proline synthesis, and influenced chlorophyll concentration; the effects were more severe under the combined heat + drought stress. ODAP content in seeds ranged from 0.06 to 0.30% under no-stress conditions. However, under heat stress, there was a significant increase of 33% in ODAP content, and under combined stress (heat + drought), the increase reached 83%. Crude protein content ranged from 15.64 to 28.67% among no stress plants and decreased significantly by 23% under heat stress and by 36% under combined stress. The findings of this study also indicated substantial reductions in growth and grain yield traits under both heat stress and combined heat + drought stress. Six accessions namely *IG 66026*, *IG 65018*, *IG 65687*, *IG 118511*, *IG 64931*, and *IG65273* were identified as having the most favorable combination of yield, protein content, and seed ODAP levels across all conditions. ODAP content in these six accessions varied from 0.07 to 0.11% under no stress and remained at moderate levels during both heat stress (0.09–0.14%) and combined stress (0.11–0.17%). *IG 66026* was identified as the most stable genotype under drought and heat stress conditions with high protein content, and low ODAP content. By identifying those promising accessions, our results have established a basis for forthcoming grasspea breeding initiatives while paving the way for future research exploration into the fundamental mechanisms driving ODAP variation in the presence of both heat and drought stress conditions.

## 1. Introduction

The impacts of climate change are increasingly threatening agricultural sustainability and food security [1]. Particularly, the crops that rely on rainfed cultivation are highly vulnerable to heat and drought stress, affecting plant growth and development [2,3,4]. In addition, the global population is increasing and is expected to reach its peak of approximately 10.9 billion by the end of the 21st century [5]. The majority of this population growth is expected to occur in developing countries, which already face challenges related to malnutrition caused by protein and micronutrient deficiencies [5,6]. In response to these challenges, there has been a growing emphasis on exploring the genetic potential of underutilized crops that perform well with minimal inputs such as fertilizer and irrigation under climate change scenarios [7]. Among these crops, grasspea (*Lathyrus sativus* L.) has garnered increasing attention as a promising candidate [8,9,10,11].

Grasspea, an annual cool-season crop from the Fabaceae family, is recognized as one of the most resilient legume crops globally [12,13]. Its cultivation can be traced back to the early Neolithic period, as evidenced by archaeological findings in the Balkan Peninsula [14]. Grasspea holds substantial economic and ecological value as a food, feed, and fodder source in South Asia and Sub-Saharan Africa. Nevertheless, its cultivation is relatively limited in Central and West Asia and North Africa (CWANA), southern Europe, and South America [15].

The genus *Lathyrus* encompasses 187 species which are the warehouse of many traits of interest for food, nutrition, and environmental security [16]. Grasspea is renowned for its ability to withstand severe drought stress, tolerate waterlogging, heat and salinity, and resist several insect pests and diseases [17,18]. It plays a key role in low-input farming systems owing to its efficient atmospheric nitrogen fixing ability reaching up to 124 kg/ha, and its adaptability to diverse soil types [19]. Likewise, its high seed protein content (up to 29.9% *w*/*w*) makes grasspea a promising addition to cereal-centric diets for poor populations [20,21,22,23]. Nevertheless, excessive consumption of grasspea causes neurolathyrism, an irreversible paralysis of the legs coming from a neurotoxin, β-N-oxalyl-L-α, β- diaminopropionic acid (β-ODAP) [24,25]. The presence of this plant toxin in both wild and most cultivated forms of grasspea has limited its potential and caused a setback to its cultivation [26]. Consequently, breeding programs have focused on identifying low β-ODAP varieties [27]. Meanwhile, β-ODAP levels in grasspea plants and seeds show variability across different locations, influenced by genotype, environmental factors, and their complex interactions [9,22,28,29,30,31]. In light of this, Some studies recorded a positive association between high levels of β-ODAP biosynthesis and water stress in grasspea [32,33,34,35].

The impact of heat and drought stress on plant growth and productivity is extensively documented. When heat and drought stresses coincide with gametogenesis, flowering, and anthesis, both pollen and ovules can be negatively affected, leading to reduced pollen viability, impaired pollen development, and increased sterility [36]. Additionally, seed filling represents another critical stage involving intricate biochemical processes for carbohydrate, protein, and lipid synthesis, and is highly susceptible to heat and drought stresses [37,38,39]. Nevertheless, very little information is available concerning the combined effects of heat and drought, despite the evident coupling between these two stressors and their detrimental implications for crop growth and productivity [40,41,42]. Recent studies have illuminated the adverse consequences of these conditions on cereals including wheat [43], and legumes including lentils [44,45,46,47,48], and chickpeas [37]; however, this aspect remains unexplored in grasspea, and the mechanisms underlying its resistance remain are insufficiently investigated. Thus, our study presents a pioneering exploration into the heat stress and the interplay of drought-heat stress during reproductive and seed-filling stages on ODAP content, physiological traits, phenology, grain yield, and nutritional quality within grasspea germplasm under controlled conditions. Additionally, the identification of accessions capable of delivering superior grain yield, elevated protein content, and reduced ODAP levels under both optimal and stressful conditions hold immense importance and offer valuable insights for a grasspea breeding program. Haut du formulaire.

Hence, The primary objective of the present study was (i) to investigate the impact of heat stress and the interaction of heat + drought stress during the reproductive stage on ODAP content, physiological, phenological, nutritional, and yield-related traits in grasspea germplasm and its crop wild relatives under controlled conditions, (ii) to assess the genetic variation for the investigated traits, and (iii) to identify the germplasm with high protein and low ODAP contents under both optimal and stress conditions. 

## 2. Results

### 2.1. Analysis of Variance and Tukey’s Test

Analysis of variance (ANOVA) showed highly significant differences (*p* < 0.001) among 24 accessions for all the investigated traits, excluding seed circularity. The treatment effects of heat stress and combined heat + drought stress were also found to be significant for all the traits except for seed eccentricity and Feret’s diameter. Similarly, the genotype × treatment interaction effect was also significant for all measured traits besides total chlorophyll, 100-seed weight, harvest index, crude protein, and some seed size and shape parameters (Table 1, Table 2, Table 3 and Table 4).

### 2.2. Stress Effect on Phenology

Observations revealed that heat stress applied independently or in combination with drought stress during the pre-flowering stage accelerated phenology (Table 1). Under stress conditions, the duration between sowing and first flowering decreased by 9% and 17% under heat stress and combined heat + drought stress treatments, respectively. Likewise, the time to podding was shorter under heat (9% reduction over no stress) and combined heat + drought (17% reduction over control). Days to maturity were significantly reduced by 11% under individual heat and 18% under combined stress relative to no-stress conditions.

### 2.3. Effect on Growth and Grain Yield

The yield component experienced a significant decrease under combined heat + drought stress compared to individual heat stress (Table 2). The aboveground biomass exhibited a reduction of 46% in heat-stressed plants and a more substantial decrease of 62% under combined stress, compared to no-stress conditions. Plant height notably decreased in heat-stressed plants (28% reduction over no stress) and declined further in combined heat + drought-stressed plants (38% over no stress).

Under stress treatments, there were significant reductions in the number of filled pods and seed numbers compared to no-stress conditions. Under heat stress, the number of filled pods and seeds declined by 45% and 60%, respectively. Under the combined heat + drought stress, these reductions were more pronounced, with a decline of 61% in filled pods and 75% in seed numbers. Grain yield decreased by 69% under heat stress and it further decreased to 82% under combined heat + drought-stressed plants, compared with no stress. Additionally, individual heat stress and combined heat + drought stress reduced 100-seed weight by 19% and 21%, respectively, compared to no-stress conditions. Consequently, the harvest index was significantly affected and exhibited a reduction of 38% in heat-stressed plants and 50% under combined heat + drought conditions, compared to no-stress plants.

### 2.4. Effect on Leaf Water Status and Photosynthetic Function

Leaf temperature exhibited a significant increase of 27% in heat-stressed plants and 33% in combined heat + drought-stressed plants compared to no stressed plants (Table 3). Relative leaf water content (RLWC) decreased by 15% under heat stress and 27% under combined heat + drought stress conditions.

Leaf chlorophyll concentration also dropped under combined heat + drought stresses (58% reduction over no stress) compared to individual heat stress (37% reduction over no stress). Proline content in leaves significantly increased relative to no-stress conditions by 223% and 180% under heat stress and combined stress, respectively.

### 2.5. Effect on ODAP and Crude Protein Contents

There was a significant increase in ODAP content under heat and drought stress treatments, with a higher accumulation observed in leaves compared to seeds (Table 3). The application of combined stress treatment led to a notable increase in ODAP content in leaves by 69% and in seeds by 83%. In comparison, individual heat stress resulted in a rise of 31% in leaf ODAP content and 33% in seed ODAP content, both relative to the no-stress conditions. On the other hand, crude protein content was significantly decreased by heat stress (reduction of 23%) and combined heat + drought conditions (reduction of 36%).

### 2.6. Effect on Seed Size and Shape Parameters

The seed size and shape parameters showed slight variations under stress treatments (Table 4). When heat stress was applied either independently or in combination with drought, there were respective decreases observed in seed length (8, 9%), width (7, 9%), area (12, 14%), perimeter (8, 9%), equivalent diameter (6, 7%), circularity (5, 6%), and rugosity (4, 4%). Interestingly, seed thickness was found to increase under heat treatment (1%) and combined heat + drought (1%). However, stress treatments had no significant effect on seed eccentricity and Feret’s diameter.

### 2.7. Effect of Heat and Heat + Drought Stresses on Interrelationships among the Observed Traits

**Figure 1:**
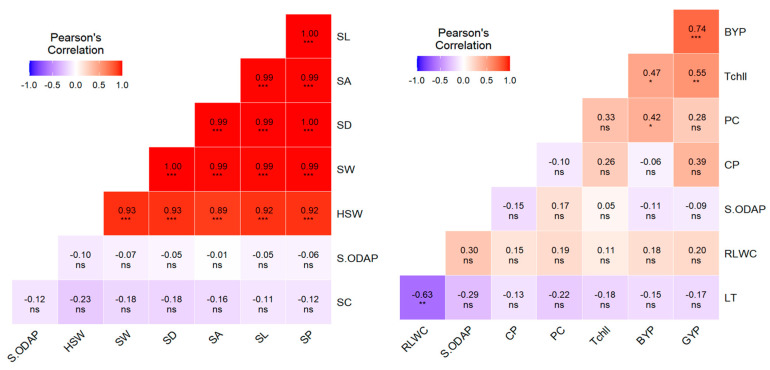
Pearson correlations between various trait combinations under no-stress conditions. *, **, ***, and ns indicate significance at 0.05, 0.01, and 0.001 probability levels, and non significance respectively.

**Figure 2:**
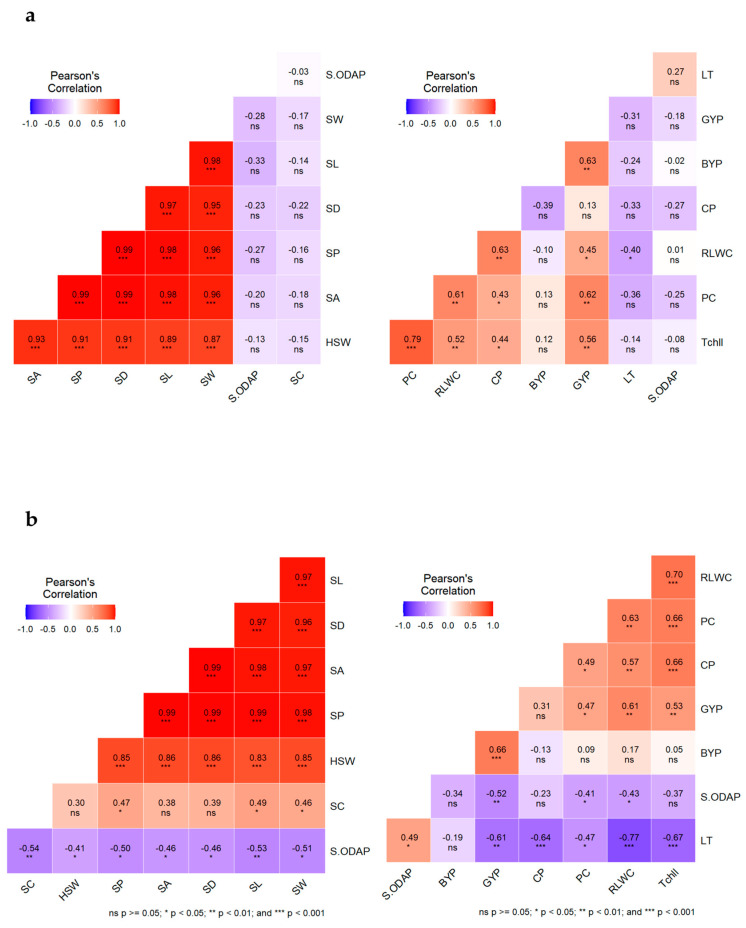
Pearson correlations between various trait combinations under heat stress (**a**), and combined heat + drought stress (**b**). *, **, ***, and ns indicate significance at 0.05, 0.01, and 0.001 probability levels, and non significance respectively.Under no stress conditions, leaf ODAP content showed a significant negative correlation with biological yield (r = −0.422, *p* < 0.05) and a positive correlation with seed ODAP content (r = 0.697, *p* < 0.01). However, under heat stress, ODAP content in leaves and seeds did not show any significant correlation with other traits of interest. Under combined heat + drought stress, seed ODAP content showed a significant positive correlation with leaf temperature (r = 0.485, *p* < 0.05) and a significant negative correlation with RLWC (r = −0.431, *p* < 0.05), proline content (r = −0.407, *p* < 0.05), filled pods (r = −0.407, *p* < 0.05), grain yield (r = −0.517, *p* < 0.01), and 100-seed weight (r = −0.410, *p* < 0.05). In no-stress conditions, a significant positive correlation was found between crude protein and number of filled pods (r = 0.457, *p* < 0.05), seed number (r = 0.547, *p* < 0.01), and harvest index (r = 0.611, *p* < 0.01). Similar correlations were observed under heat stress and combined heat + drought, except for the harvest index, which did not show a significant association under heat stress. In addition, our results revealed a negative, but not significant correlation between seed ODAP and protein content in all treatments.

Pearson’s correlation was performed between traits under no stress (Table S1), heat stress (Table S2), and combined heat + drought stress conditions (Table S3). The analysis revealed significant correlations among various trait combinations (Figure 1 and Figure 2). Significant negative correlations were observed between leaf temperature and RLWC across all the treatments (*p* < 0.05). Under heat stress, RLWC and chlorophyll concentration had significant positive correlations with filled pods (r = 0.595, *p* < 0.01; r = 0.681, *p* < 0.01), total number of pods (r = 0.582, *p* < 0.01; r = 0.597, *p* < 0.01), and grain yield (r = 0.445, *p* < 0.05; r = 0.561, *p* < 0.01). These correlations remained highly significant (*p* < 0.01) when the two stresses were combined and showed significant positive correlations with the number of unfilled pods (r = 0.447, *p* < 0.05; r = 0.475, *p* < 0.05), seed number (r = 0.617, *p* < 0.01; r = 0.677, *p* < 0.01), and harvest index (r = 0.703, *p* < 0.01; r = 0.706, *p* < 0.01). In contrast, significant positive correlations were observed between proline content and heat stress and combined heat + drought stress conditions. These associations were observed with leaf water status (r = 0.612, *p* < 0.01; r = 0.628, *p* < 0.01), chlorophyll content (r = 0.788, *p* < 0.01; r = 0.660, *p* < 0.01), and crude proteins (r = 0.430, *p* < 0.05; r = 0.494, *p* < 0.05). Furthermore, proline content showed significant correlations with certain yield traits, including filled pods (r = 0.628, *p* < 0.01; r = 0.748, *p* < 0.01), total number of pods (r = 0.514, *p* < 0.05; r = 0.619, *p* < 0.01), and grain yield (r = 0.618, *p* < 0.01; r = 0.474, *p* < 0.01).

Highly significant correlations (*p* < 0.01) were observed between 100-seed weight and some seed size and shape parameters including seed length, width, area, perimeter, and diameter in all treatments. Moreover, 100-seed weight showed a significant positive association with seed turgidity (r = 0.498, *p* < 0.05; r = 0.422, *p* < 0.05; r = 0.540, *p* < 0.01) while displaying a significant negative association with seed rugosity (r = −0.468, *p* < 0.05; r = −0.609, *p* < 0.01; r = −0.581, *p* < 0.01) under no stress, heat, and combined heat + drought stress, respectively. Under combined heat + drought stress, seed ODAP content exhibited significant negative correlations with seed length (r = −0.528, *p* < 0.01), width (r = −0.510, *p* < 0.05), area (r = −0.457, *p* < 0.05), perimeter (r = −0.498, *p* < 0.05), diameter (r = −0.466, *p* < 0.05), and seed circularity (r = −0.541, *p* < 0.01).

### 2.8. Principal Component Analysis

Principal component analysis (PCA) was conducted for each of the three treatments. Under no stress conditions, the first two components (PC1 and PC2) explained 47% of the total variability with PC1 accounting for 28% and PC2 19% (Table S4). Grain yield, number of filled pods, day to flowering, total chlorophyll, and 100-seed weight contributed significantly to PC1, while the number of unfilled pods, harvest index, number of seeds per plant and per pod, and day to maturity had the highest influence on PC2. When subjected to heat stress, PC1 and PC2 accounted for 32.4 and 15.6% of the total variation. However, when heat and drought stresses were combined, PC1 and PC2 explained 42.1% and 16.4% of the total variation, respectively. The variance in PC1 under stress conditions was mainly due to relative leaf water content, total chlorophyll, and the number of total and filled pods, while biological yield and 100-seed weight were the most contributing traits to PC2 under stress treatments.

**Figure 3:**
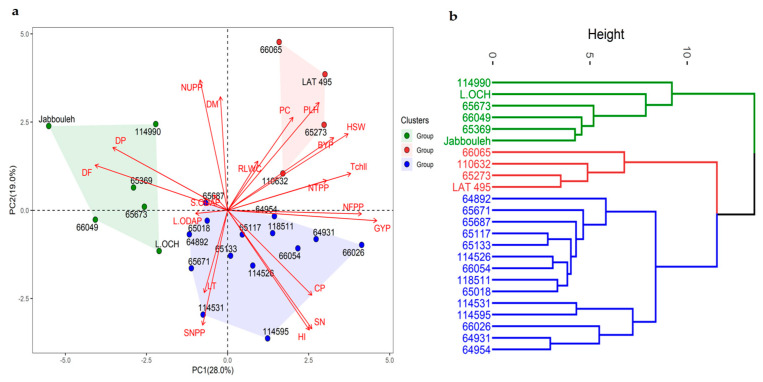
Principal component analysis (**a**), hierarchical clustering (**b**), and various traits contributing to the variability under no stress.

Based on the Biplot of PCA, the examined accessions were categorized into three distinct groups. Under no stress conditions (Figure 3), group 1 consisted of five accessions characterized by the lowest number of filled pods (9.47), grain yield (2.15 g), and 100-seed weight (8.73 g) (Table S5). These accessions exhibited the highest seed ODAP content (0.16% DW). In contrast, group 2 included accessions with the highest number of filled pods (11.94), grain yield (4.10 g), and 100-seed weight (16.50 g). Their seeds displayed moderate ODAP content (0.14% DW). Cluster 3 comprised four accessions having a moderate number of filled pods (11.38), grain yield (3.17 g), and 100-seed weight (10.91 g), along with low and moderate seed ODAP content (0.11% DW).

**Figure 4:**
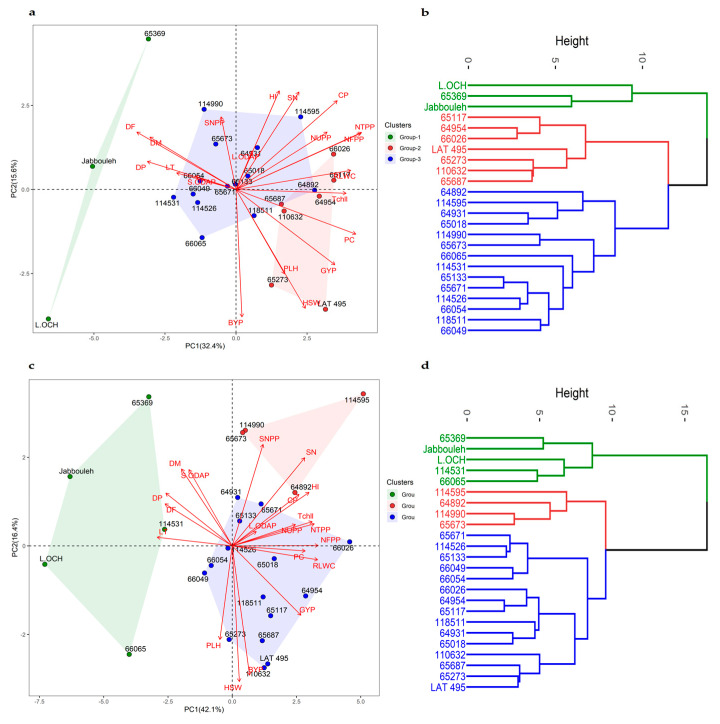
Principal component analysis, hierarchical clustering, and various traits contributing to the variability under heat stress (**a**,**b**), and combined heat + drought stress conditions (**c**,**d**).

Under heat stress conditions (Figure 4), three accessions belonging to group 1 exhibited late flowering (53.33 DAS) and maturity (80.00 DAS), as well as the low relative water content (59.54%), chlorophyll concentration (7.26 mg/g DW), grain yield (0.73 g), and crude protein content (13.68%). Notably, this group displayed the highest ODAP content in their seed tissues (0.18% DW). However, group 2 consisted of accessions characterized by early flowering (40.10 DAS) and maturity (73.62 DAS), along with high relative water content (72.26%), chlorophyll concentration (12.68 mg/g DW), grain yield (1.44 g), and crude proteins (18.03%). These accessions recorded the lowest seed ODAP content (0.14% DW). Lastly, accessions categorized under group 3 exhibited medium late flowering (41.32 DAS) and maturity (74.82 DAS), accompanied by medium relative water content (67.81%), chlorophyll concentration (9.40 mg/g DW), grain yield (0.79 g), crude protein (17.65%), and seed ODAP content (0.17% DW).

Under combined heat + drought (Figure 4), group 1 consisting of five accessions exhibited late flowering (45.57 DAS), and low grain yield (0.35 g) and crude protein (12.63%). This group was distinguished by high seed ODAP content (0.28% DW). Group 2 had four accessions that flowered earlier (35.83 DAS), with moderate grain yield (0.56 g), protein (15.68%), and seed ODAP (0.28% DW) contents. In group 3, promising accessions were identified with medium flowering dates (37.17 DAS), high grain yield (0.65 g), and moderate levels of crude proteins (14.47%) and seed ODAP content (0.18% DW).

### 2.9. Hierarchical Cluster Analysis

Hierarchical cluster analysis categorized 24 accessions into three groups based on grain yield, protein content, and seed ODAP content. The mean values of these traits for each cluster are provided in Table 5. Under no stress conditions, the first cluster consisted of seven accessions characterized by high grain yield, protein content, and ODAP content. The second group comprised 12 accessions with moderate grain yield, moderate seed ODAP, and high protein content. The third cluster contained five accessions displaying high grain yield, low seed ODAP content, and high protein levels compared with other groups. When subjected to heat stress conditions, the first cluster revealed eight accessions with high grain yield, moderate ODAP content, and high protein content relative to the second cluster which included 12 accessions characterized by moderate yield, moderate seed ODAP level, and high protein content. The remaining accessions exhibited moderate grain yield, high ODAP content, and moderate protein levels. Under combined heat + drought stress, the first cluster consisted of six accessions showing high yield, moderate ODAP content, and moderate crude proteins. The second cluster grouped 11 accessions with low yield, high ODAP content, and moderate protein levels. The third cluster comprised accessions with moderate yield, ODAP content, and protein levels.

### 2.10. Identification of Promising Germplasm

Our study identified six stable grasspea germplasm with high grain yield and protein content, along with low or moderate ODAP content under no stress, heat stress, and combined heat + drought stress environments (Table 6). Among the six accessions, three belonged to cultivated species, while the remaining three accessions represented three wild species (*Lathyrus tingitanus*, *Lathyrus inconspicuus*, and *Lathyrus annuus*). When comparing these accessions under no stress conditions, *IG 66026*, *IG 65018*, and *IG 65687* showed low ODAP content (0.07% DW). On the other hand, *IG 66026* and *IG 64931* exhibited high protein content under all treatments. Furthermore, *IG 66026* gave the highest grain yield under no stress conditions followed by *IG 65273*, whereas *IG 66026* and *IG 65687* showed the highest grain yield under heat as well as under combined heat + drought treatments. *IG 65018* had the lowest ODAP content under stress conditions.

## 3. Discussion

Our study revealed that heat stress, either alone or in combination with drought stress at the pre-flowering stage had a significant impact on phenology, physiology, yield components, and seed quality in *Lathyrus* germplasm. Despite being known to be adapted to dry areas [10,12,17,18], high temperatures (38 °C/24 °C) and longer periods of water deficit exposure during the reproductive and seed-filling stages severely affected water relations and photosynthesis efficiency in grasspea. The results showed a significant reduction in the time and duration of flowering, podding, and maturity under stress treatments. This phenology acceleration resulted in the rapid development of plants to escape terminal heat and drought. The same adaptation mechanism was noted in grasspea [49], common bean [50], chickpea [51], and lentil [46,48].

Our research suggests that elevated temperatures can lead to an increase in leaf temperature, which exhibited a strong negative correlation with relative leaf water content across all treatments. Despite regular irrigation of pots to maintain field capacity, heat-stressed plants experienced a significant reduction in leaf water content. This implies that temperature and relative humidity differences between no-stress and heat-stress conditions could be responsible for a significant increase in transpiration, leading to leaf dehydration and ultimately causing a decrease in water content in heat-stressed plants. Under stress conditions, a decrease in chlorophyll concentration was observed across all accessions. This effect is likely the result of chlorophyll biosynthesis inhibition or its increased deterioration caused by photooxidation and lipid peroxidation of chloroplast membranes [52,53,54]. The combined effect of heat + drought stress further intensified this decline which might impact various components of the photosynthetic machinery, including the D1, D2, and CP47 proteins of PSII, as well as the activity of RuBisCo, which is a crucial enzyme for carbon fixation [55]. Similar observations on chlorophyll content have been reported previously in grasspea [56,57,58] exposed to drought stress, lentil [44,45] and chickpea [37] under heat and combined heat + drought stress. Several stress-tolerant grasspea genotypes [56,58] and other crops [59,60] have been found to accumulate proline in response to water loss. Our findings are consistent with those results, as a higher concentration of proline under stressful conditions was generally observed. Proline serves as a compatible osmolyte, which helps to decrease osmotic potential, thereby maintaining cell turgidity without interfering with protein synthesis [61,62]. In addition, proline has been found to play several other important roles, including protecting cellular structures, proteins, and membrane integrity, reducing oxidative damage to lipid membranes, scavenging reactive oxygen species (ROS), and stabilizing redox potential [63,64,65]. The lower proline content observed in some of the accessions under stress conditions compared to the no stress may be due to its degradation by high temperatures, incorporation into proteins, or inhibition of its biosynthesis [66].

Heat and combined heat + drought stress significantly decreased growth and yield in grasspea accessions. The decline of plant biomass and plant height can be associated with the stress effect that induced early maturity and limited growth-related metabolism. In the case of heat + drought stress, the withholding of water intensified the reduction in plant height, consistent with previous findings that highlighted a strong correlation between plant height and rainfall in grasspea [10]. In this study, the relative leaf water content and chlorophyll concentration have shown a positive correlation with the number of filled pods, total number of pods, and grain yield under stress treatments. Therefore, our findings indicated that yield reduction might be linked to water deficit, photosynthetic damage, and earlier maturity as reported in grasspea water-stressed plants [67], lentil [44], and chickpea [37] under heat and combined heat + drought stress. In addition, the drop in pod and seed numbers, along with grain yield, could be attributed to the adverse effects of individual heat and combined stress on flowers and pod production [67], as well as on pollen viability and germination [49]. Gusmao et al. [49] and Kong et al. [67] established a connection between the substantial yield decline of grasspea during water deficit and the decrease in flower production, coupled with an increase in the rates of flower, pod, and ovule abortion. Studies have shown [68] that seed-filling duration in grasspea is highly sensitive to climatic factors. Thus, the reduction in grain yield is possibly due to the decline in the supply of sucrose from the leaves to developing seeds and pods along with the inhibition of enzymes involved in starch synthesis, as suggested by Kaushal et al. [69], and Awasthi et al. [37]. Additionally, under heat and heat + drought stress conditions, a decrease in the harvest index was also observed. A similar drastic reduction in growth and yield components was reported in grasspea under water deficit [49,67,70], and lentil exposed to heat [3,47,71] and combined heat and drought [44,45,46,48,72].

Several authors [22,35,49] reported consistency in seed size across various grasspea genotypes under both controlled and field conditions, indicating an adaptive response to the specific studied environments. However, in this study, seed area and perimeter moderately decreased under stress conditions compared to no stress, while seed length, width, diameter, circularity, and rugosity showed slight changes. On the other hand, there was no significant effect on seed eccentricity and Feret’s diameter. This reduction in seed size and shape parameters is consistent with previous findings in grasspea [67], and lentil, [45,47] where reduced seed size under stress conditions was associated with seed-filling alteration and led to a decline in hundred seed weight.

The effect of heat and heat + drought stress on ODAP content was highly significant in the studied accessions. This neurotoxin increased in seeds and leaves under heat, and when the two stresses were combined, it increased even further. Being the predominant amino acid within grasspea seeds, β-ODAP accumulation could be linked to the ability of grasspea to endure water stress, making it a valuable species in arid regions [73]. In light of this, The buildup of β-ODAP in grasspea plants and seeds during drought stress may stem from disrupted nitrogen assimilation, causing elevated asparagine production [74]. Asparagine is a primary precursor for the isoxazoline ring of β-(isoxazolin-5-on-2-yl) alanine (BIA), which subsequently acts as the precursor for β-ODAP [75]. Accordingly, Xiong et al. [76], and Zhou et al. [77] detected the correlation between ODAP and ABA signaling, polyamine metabolism, and radical oxygen species (ROS) scavenging, involved in the signaling pathway of plant response to water stress. However, the divergent patterns observed between proline and ODAP in our study contradict the hypothesis that β-ODAP might function as a stress-induced compatible osmolyte. Leaves exhibited a higher level of ODAP compared to seeds in all treatments when different parts of the plant were compared. Previous research indicated that grasspea contained β-ODAP in all tissues [78,79,80], and its accumulation varied according to the stage of plant growth [80]. The correlation between ODAP content in seeds and leaves was positive and significant under no stress conditions but became non-significant under heat and combined heat + drought stress. These findings align with previous research that highlights the shifting concentration of ODAP from vegetative leaf tips to reproductive stage sinks such as developing seeds during plant growth [81].

Previous studies have demonstrated a similar outcome in grasspea, wherein the buildup of ODAP was detected in the leaves of 15-day-old seedlings [32], and seeds of growing plants [33,34,35] under water deficit conditions. Several authors reported that genetic and environmental factors, particularly heat and drought have an impact on the concentration of ODAP in grasspea [17,28,29,30,31]. Conversely, other studies examining grasspea under drought stress have shown either insignificant differences or minimal variations in ODAP content when compared with no-stress conditions [33,49,82]. The negative correlation among seed ODAP, grain yield, and seed size was particularly significant under combined heat + drought conditions, indicating that larger seed size and higher seed numbers may dilute available β-ODAP. Consequently, the selection of germplasm with high yield and large seed could be advantageous in developing varieties with reduced levels of β-ODAP [33,82].

Furthermore, heat and drought can negatively affect the nutritional quality of plants by reducing the accumulation of proteins. Our study showed exposure to high temperatures resulted in a decrease in total nitrogen levels, leading to a decline in protein content. Recently, Choukri et al. [47] attributed the decrease in crude protein to the inhibition of protein synthesis caused by heat stress. Our findings indicated that the combined effect of heat and drought had a more pronounced impact on reducing proteins. This could be attributed to the relationship between water availability and the ability of root nodules to fix nitrogen [83,84]. When both stresses are present, increased water loss can significantly hinder this function. Similar results have been reported in lentil [47,48] and chickpea [37]. In contrast, Boukecha et al. [70] and Yang et al. [34] observed increased protein content in grasspea when subjected to drought conditions.

Principal component analysis was employed to differentiate the evaluated *Lathyrus* accessions. Chlorophyll concentration and number of filled pods were prominent traits contributing to variation across all treatments. Under stress conditions, relative leaf water content and total pod number played a more significant role. Multivariate analysis coupled with hierarchical cluster analysis was useful in grouping accessions exhibiting similar responses. However, a further cluster analysis was conducted on grain yield, seed ODAP, and crude protein to specifically address the main concerns in grasspea breeding [28,33]. The observed negative association between seed ODAP content and both grain yield and crude proteins presents an opportunity to achieve a desirable combination of low ODAP, high grain yield, and high protein content within the germplasm. Thus, six promising accessions (*IG 66026*, *IG 65018*, *IG 65687*, *IG 118511*, *IG 64931*, and *IG 65273*) were identified as highly promising for future grasspea breeding improvement. The ODAP levels in these promising accessions ranged from 0.07 to 0.11% under no-stress conditions and remained at moderate levels during heat stress (0.09–0.14), and combined heat + drought stress (0.11–0.17). Notably, *IG 66026* emerged as the most stable genotype exhibiting the most favorable combination of yield, protein content, and seed ODAP levels across all conditions.

## 4. Materials and Methods

### 4.1. Plant Material

A set of 24 accessions representing eleven *Lathyrus* species was selected from a diversity panel of 435 germplasm accessions based on the ODAP content, grain yield, and biomass as selection criteria. These accessions were obtained from the ICARDA genebank, Rabat, Morocco. The details about these accessions are given in Table S6.

### 4.2. Growth Conditions and Experimental Treatment

The experiment was conducted in two growth chambers at ICARDA-Rabat, Morocco with one chamber designated as no stress (A), and the other as stress treatments (B). Plastic pots with a diameter of 15 cm and a height of 20 cm were filled with a mixture of sandy loam soil and compost garden soil in a 2:2 (*w*/*w*) ratio, totaling 1.5 kg of soil. The alpha lattice design with three replications was used to arrange the 24 *Lathyrus* accessions. In each pot, five seeds were sown at a depth of 2 cm on 29 March 2022. After germination, the number of seedlings was reduced to four per pot, ensuring the same plant density. The plants were adequately irrigated to maintain approximately 100% field capacity in both growth chambers. The average day/night temperatures and relative humidities in the chambers were 28 °C/18 °C and 86.2/58.1%, respectively. Stress was imposed during the pre-flowering stage (one month after germination), in the growth chamber labeled as B. This involved subjecting the plants to high temperatures, with mean day and night temperatures reaching up to 38 °C/24 °C, which resulted in a decrease of the average day/night relative humidity to 70.8/45.8%. For the pots experiencing combined heat + drought stress, irrigation was withheld, while the heat-stressed plants in the same chamber continued to receive water to maintain field capacity. These conditions were maintained until the plants reached maturity.

### 4.3. Data Collection

For each of the three treatments, phenological traits including days to first flowering, days to first podding, and physiological maturity were recorded on a whole plot basis. Fifteen days after the initiation of stress (during the peak-flowering stage), leaflets were collected at 11:00 AM from the second and third branches from the top to assess chlorophyll content and relative leaf water content. Leaf temperature was measured by taking three observations from different positions on the marked leaves, which were then combined and calculated as an average per leaf per plant. Proline content was determined by collecting leaflets, preserving them in liquid nitrogen, and subsequently freeze-drying and grinding them.

At the maturity stage, the plants were cut at the soil level. Observations on plant height (cm), total number of seeds, pods, filled pods, and unfilled pods, biological yield (g), grain yield (g), and number of seeds per pod were recorded on three plants per accession in three replications for each treatment. The plants utilized for physiological analysis were not included in the measurement of yield traits. After the harvest, pods were threshed, and cleaned seeds were used to determine quality traits such as 100-seed weight, seed size, seed shape, and ODAP and protein contents.

### 4.4. Relative Leaf Water Content (RLWC)

The relative leaf water content (RLWC) was estimated using the method described by Barrs and Weatherley [85]. Fresh leaflet samples, consisting of 4–5 leaflets from the uppermost branch during the peak flowering stage, were collected between 11:00 and 11:30 AM from accessions subjected to no stress, heat stress, and combined heat + drought stress conditions. The collected leaflets were initially weighed to determine their fresh weight. Subsequently, leaflets were placed in distilled water in petri dishes and left overnight. After removing the leaves from the water and allowing them to surface dry with blotters, they were re-weighed to obtain the turgid weight. The leaf samples were then dried in an oven (Binder, model ED 23, Germany) at 80 °C for 24 h and weighed once again to obtain the dry weight. The RLWC was calculated using the following formula:

(1) $$\mathrm{RLWC}\;(\%)=\frac{FW-DW}{TW-DW}\;\times \;100$$

where FW is the fresh weight (g), TW is the turgid weight (g), and DW is the dry weight (g).

### 4.5. Leaf Temperature of Plants

Leaf temperature of the fully expanded leaves of no stressed, heat stressed, and combined heat + drought stressed plants was recorded using an infrared sensor (Fluke 561 Infrared Thermometer, HVAC Pro model).

### 4.6. Chlorophyll Concentration

To measure chlorophyll concentration, the experimental procedure followed the principle of Arnon’s simultaneous equation [86]. Fresh leaflets (0.1 g) were extracted using 80% acetone. The resulting extract was subjected to centrifugation at a speed of 5701.8× *g* for 10 min. The chlorophyll content was determined by measuring the optical density (OD) using a UV Visible spectrophotometer (T80 series, pg instruments, UK). Specifically, the absorbance of the supernatant was read at wavelengths of 645 nm and 663 nm. The measurement of total chlorophyll was obtained by comparing it against a blank consisting of 80% acetone [37]. The following equations were utilized to calculate the concentrations of chlorophyll a, chlorophyll b, and total chlorophyll, respectively:

(2) $$\mathrm{Chl}\;a=12.9\;(A_{663})-2.69\;(A_{645})\;\times \;\frac{V}{1000\times W}$$

(3) $$\mathrm{Chl}\;b=22.9\;(A_{645})-4.68\;(A_{663})\;\times \;\frac{V}{1000\times W}$$

Total chl = Chl a + Chl b(4)

where *V* is the volume of 80% acetone added (mL), *W* is tissue weight (g), A_663_ is the absorbance at 663 nm and A_645_ is the absorbance at 645 nm.

To prevent any influence from variations in water content, chlorophyll was extracted from fresh leaves and subsequently expressed on a dry weight (DW) basis.

### 4.7. Leaf Free Proline Content

The free proline content was determined using the method developed by Troll and Lindsley [87]. Leaf samples were freeze-dried and homogenized in 10 mL of 3% sulfosalicylic acid. After centrifugation at 4000 rpm for 5 min, 2 mL of the supernatant was mixed with 2 mL of acid ninhydrin and 2 mL of glacial acetic acid in a test tube. The reaction mixture was incubated at 100 °C for 1 h and then cooled in an ice bath. To extract the chromophore, 4 mL of toluene was added and vigorously mixed. The toluene phase containing the chromophore was separated from the aqueous phase and warmed to room temperature before measuring the absorbance at 520 nm. Toluene was used as a blank for calibration. 

### 4.8. Seed Size and Shape Parameters

Seed parameters, including seed area, perimeter, length, width, circularity, diameter, thickness, rugosity, and 100-seed weight, were measured using image analysis facilitated by a high-speed seed counting device called OptoAgri2 (Optomachine, France). For each treatment, measurements were collected from three plants per accession in three replications.

### 4.9. ODAP Content in Leaves and Seeds

Total α/β-L-ODAP content was estimated using the spectrophotometric method developed by Rao et al. [88], adapted by Briggs et al. [89], and further optimized by Emmrich et al. [78]. Ground samples of seeds and leaves were separately subjected to ODAP extraction by adding 60% ethanol and incubating with shaking at room temperature for 22 h. The samples were centrifuged at 16,250× *g* for 10 min. Soon after, a 96-well microtiter plate was prepared, with 160 μL of 3 M potassium hydroxide (KOH) solution and 80 μL of aliquot. The plate was then incubated in a water bath at 95 °C for 30 min and subsequently submerged in water at room temperature.

For the analysis, a reagent buffer consisting of o-phthalaldehyde/tetraborate, as described by Emmrich et al. [78], was prepared. In a separate 96-well microtiter plate with a clear flat bottom, 30 μL of the hydrolysate was mixed with 220 μL of the OPA/tetraborate buffer. Simultaneously, another plate was loaded with 20 μL of 3 M potassium hydroxide solution (KOH) and 10 μL of the non-hydrolyzed supernatant from the extraction, followed by 220 μL of the OPA/tetraborate buffer. The mixture in each well was kept for 30 min for incubation at room temperature before the absorbance was read at 420 nm using an optical plate reader (Biotek, 800TSMB, Agilent, USA). To express the ODAP content percentage, a series of standards were included with each plate of samples to ensure accurate quantification of ODAP.

(5) $$ODAP=\frac{\left(A_{hyd}-A_{nonhyd}\right)\times V_{ext}}{m_{sample}\times a_{standard}}\;\times \;100$$

where ODAP is the concentration of total α/β-L-ODAP, A*_hyd_* is the absorbance reading of hydrolyzed sample, A*_nonhyd_* is the absorbance reading of non-hydrolyzed sample, V_ext_ is the volume of extraction buffer in mL, m_sample_ is the mass of the seed meal sample in mg, and *a_standard_* is the slope of the standard curve.

### 4.10. Protein Content

Crude protein content was estimated using the Kjeldahl method [90]. Ground seed samples were subjected to digestion by heating in a digestion block (QBlock series, Ontario, Canada) at 300 °C for 5 h in the presence of sulfuric acid, selenium, and salicylic acid. Once the digestion process was completed, the digest was treated with 5.5 mL of the buffer solution, 4 mL of sodium nitroprusside, and 2 mL of sodium hypochlorite. The mixture was then incubated in the dark at 37 °C for 15 min before the absorbance was measured at 650 nm. To determine the protein content, the nitrogen content was converted by multiplying it by the conversion factor of 6.25.

### 4.11. Statistical Analysis

The summary data included range and mean values with standard deviation along with analysis of variance was conducted using the General Linear Model (GLM). Post-hoc comparisons of mean values were performed using Tukey’s test. The relationships between traits were assessed using the Pearson correlation coefficient (r) using the *metan* package in R version 4.1.3 and RStudio version 1.3.31093 [91]. Principal component analysis was carried out using the *Factoextra* [92] and *FactoMineR* [93] packages in R version 4.1.3 and RStudio version 2022.02.3 + 492. Hierarchical cluster analysis was performed using Ward’s squared Euclidean distance method with the *dendextend* R package [94].

## 5. Conclusions

Grasspea has untapped potential as a resilient crop, but the presence of the toxin β-ODAP has hindered its expansion. In contrast to other legume crops, the effect of heat and combined heat + drought stress on grasspea remains insufficiently evaluated. For that reason, we evaluated 24 *Lathyrus* accessions representing 11 species under heat and combined heat + drought stress to assess ODAP levels, phenology, physiology, yield, and quality. By identifying low ODAP germplasm with high yield and protein content under heat and drought stress conditions, we have laid the foundation for future breeding efforts. Our findings highlight the significant impact of heat and heat + drought stress on physiology, yield, and nutritional quality with a highly detrimental effect of combined stress. Adaptation mechanisms such as accelerated phenology and proline accumulation were observed. Our results demonstrated that ODAP content was significantly influenced by the genotype, treatment, and their interactions. To deepen our understanding, this effect will be explored by incorporating a wider range of genotypes under field conditions and exploring the genotypic data to identify genes or QTL(s) involved in resistance mechanisms and ODAP synthesis. Furthermore, underlying mechanisms for ODAP variation require further investigation across diverse environments to provide essential insights into addressing the challenges associated with ODAP and harnessing the full potential of grasspea as a valuable and resilient crop.

## Figures and Tables

**Table 1:** Range, mean ± SD, and analysis of variance for phenological traits under no stress, heat, and combined heat + drought stress.

Trait	Treatment	Range	Mean ± SD	Genotype Effect	Treatment Effect	G × T Effect	Error	R^2^
DF (DAS)	No stress	39.33–63.67	46.74 ± 6.79	**	**	**	2.04	0.98
	H	36.00–60.00	42.47 ± 5.59					
	H + D	31.33–58.00	38.69 ± 5.88					
DP (DAS)	No stress	47.33–71.00	54.93 ± 6.71	**	**	**	2.88	0.97
	H	44.00–67.00	49.75 ± 5.98					
	H + D	40.67–64.00	45.42 ± 5.48					
DM (DAS)	No stress	77.50–92.00	84.42 ± 3.86	**	**	**	4.52	0.95
	H	70.00–83.00	75.12 ± 3.56					
	H + D	66.00–81.00	69.06 ± 3.68					

** indicate significance at 0.001 probability levels. DF, days to first flowering; DP, days to first podding; DM, days to physiological maturity; DAS, days after sowing; H, individual heat; H + D, combined heat + drought; G, Genotype; T, Treatment.

**Table 2:** Range, mean ± SD, and analysis of variance for the aboveground biomass, plant height, and yield related traits under no stress, individual heat, and combined heat + drought stress.

Trait	Treatment	Range	Mean ± SD	Genotype Effect	Treatment Effect	G × T Effect	Error	R^2^
BYP (g)	No stress	4.27–12.41	7.23 ± 2.38	**	**	**	1.4	0.88
	H	1.40–7.21	3.91 ± 1.50					
	H + D	1.71–4.36	2.72 ± 0.72					
PLH (cm)	No stress	30.67–91.33	50.76 ± 16.20	**	**	**	51	0.85
	H	20.33–59.00	36.34 ± 7.5					
	H + D	23.80–41.50	31.72 ± 5.37					
NFPP	No stress	7.00–13.67	11.08 ± 1.94	**	**	**	0.5	0.97
	H	3.00–7.44	6.05 ± 0.84					
	H + D	2.00–5.28	4.36 ± 0.78					
NUPP	No stress	4.00–6.67	4.80 ± 0.7	**	**	**	0.3	0.72
	H	4.00–7.44	5.56 ± 0.58					
	H + D	3.00–7.17	5.39 ± 0.78					
NTPP	No stress	11.22–18.89	15.76 ± 2.23	**	**	**	0.9	0.94
	H	8.00–14.06	11.66 ± 1.12					
	H + D	5.00–11.67	9.62 ± 1.46					
SN	No stress	17.60–40.43	26.94 ± 6.55	**	**	**	14	0.92
	H	7.06–15.35	10.68 ± 2.20					
	H + D	3.00–12.92	6.73 ± 2.29					
SNPP	No stress	1.54–4.19	2.45 ± 0.57	**	**	*	0.2	0.77
	H	1.06–2.25	1.76 ± 0.31					
	H + D	0.90–2.46	1.55 ± 0.43					
GYP (g)	No stress	1.39–5.53	3.11 ± 1.08	**	**	**	0.3	0.92
	H	0.59–1.65	0.97 ± 0.34					
	H + D	0.30–0.97	0.57 ± 0.20					
HSW (g)	No stress	7.43–18.72	11.39 ± 2.99	**	**	NS	4.5	0.81
	H	5.45–21.27	9.22 ± 3.60					
	H + D	5.00–17.44	9.04 ± 3.22					
HI (%)	No stress	26.03–59.38	43.81 ± 9.23	**	**	NS	61	0.79
	H	11.10–47.50	27.11 ± 6.79					
	H + D	9.59–32.87	21.99 ± 5.59					

*, **, NS indicate significance at 0.05 and 0.001 probability levels, and non-significant, respectively. BYP, biological yield plant^−1^; PLH, plant height; NFPP, number of filled pods plant^−1^; NUPP, number of unfilled pods plant^−1^; NTPP, number of total pods plant^−1^; GYP, grain yield plant^−1^; SN, seed number plant^−1^; SNPP, seed number pod^−1^; HSW, 100-seed weight; HI, harvest index; H, individual heat, H + D, combined heat + drought; G, Genotype; T, Treatment.

**Table 3:** Range, mean ±SD, and analysis of variance for the physiological traits, ODAP content, and crude protein under no stress, individual heat, and combined heat + drought stress.

Trait	Treatment	Range	Mean ± SD	Genotype Effect	Treatment Effect	G × T Effect	Error	R^2^
LT (°C)	No stress	25.76–28.98	27.35 ± 0.77	**	**	**	0.61	0.98
	H	32.26–36.69	34.61 ± 1.18					
	H + D	33.83–38.96	36.50 ± 1.53					
RLWC (%)	No stress	73.30–86.73	80.43 ± 4.03	**	**	*	30.49	0.88
	H	55.13–76.78	68.07 ± 5.44					
	H + D	44.78–69.20	58.86 ± 6.46					
Tchll (mg g−1 DW)	No stress	13.45–17.68	16.03 ± 1.32	**	**	NS	3.22	0.92
	H	6.31–14.79	10.09 ± 2.55					
	H + D	3.06–11.54	6.69 ± 2.00					
PC (µmol g^−1^ DW)	No stress	1.90–7.62	4.34 ± 1.68	**	**	**	5.76	0.95
	H	2.76–26.10	14.02 ± 6.31					
	H + D	1.52–26.80	12.15 ± 7.06					
S-ODAP (% DW)	No stress	0.06–0.30	0.12 ± 0.06	**	**	*	0.001	0.94
	H	0.09–0.34	0.16 ± 0.06					
	H + D	0.11–0.35	0.22 ± 0.06					
L-ODAP (% DW)	No stress	0.15–0.49	0.26 ± 0.07	**	**	**	0.002	0.92
	H	0.22–0.57	0.34 ± 0.11					
	H + D	0.27–0.81	0.44 ± 0.14					
CP (%)	No stress	15.64–28.67	22.35 ± 3.11	**	**	NS	2.414	0.94
	H	10.10–23.37	17.26 ± 2.71					
	H + D	9.75–19.78	14.28 ± 1.76					

*, **, NS indicate significance at 0.05 and 0.001 probability levels, and non-significant, respectively. LT, leaf temperature; RLWC, relative leaf water content; Tchll, total chlorophyll; PC, proline content; S-ODAP, seed ODAP content; L-ODAP, leaf ODAP content; CP, crude protein.; H, individual heat, H + D, combined heat + drought; G, Genotype; T, Treatment.

**Table 4:** Range, mean ±SD, and analysis of variance for seed size and shape parameters under no stress, individual heat, and combined heat + drought stress.

Trait	Treatment	Range	Mean ± SD	Genotype Effect	Treatment Effect	G × T Effect	Error	R2
SL (mm)	No stress	5.75–10.53	6.74 ± 1.06	**	**	NS	0.27	0.87
	H	4.64–9.36	6.17 ± 1.02					
	H + D	4.53–8.83	6.14 ± 1.05					
SW (mm)	No stress	4.82–8.40	5.68 ± 0.78	**	**	*	0.15	0.87
	H	4.16–7.71	5.29 ± 0.78					
	H + D	3.87–7.37	5.19 ± 0.84					
SA (mm2)	No stress	20.59–67.50	29.14 ± 9.95	**	**	NS	18.92	0.88
	H	14.20–56.09	25.51 ± 8.52					
	H + D	13.15–50.92	25.16 ± 8.73					
SP (mm)	No stress	20.64–37.978	24.48 ± 3.80	**	**	NS	3.59	0.87
	H	16.90–34.10	22.63 ± 3.56					
	H + D	15.83–32.70	22.30 ± 3.95					
SD (mm)	No stress	5.05–9.23	5.99 ± 0.91	**	**	NS	0.2	0.63
	H	4.25–8.42	5.61 ± 0.86					
	H + D	4.09–8.00	5.55 ± 0.91					
SE (mm)	No stress	0.39–0.54	0.46 ± 0.04	**	NS	**	0.002	0.74
	H	0.31–0.61	0.45 ± 0.08					
	H + D	0.30–0.62	0.47 ± 0.08					
SFD	No stress	1.09–1.23	1.15 ± 0.04	**	NS	NS	0.003	0.62
	H	1.07–1.28	1.14 ± 0.08					
	H + D	1.05–1.30	1.14 ± 0.06					
SC	No stress	1.02–1.49	1.12 ± 0.11	**	*	NS	0.019	0.36
	H	1.02–1.40	1.06 ± 0.07					
	H + D	1.00–1.08	1.05 ± 0.02					
ST	No stress	0.72–0.76	0.74 ± 0.01	NS	**	NS	0.0002	0.64
	H	0.72–0.78	0.75 ± 0.02					
	H + D	0.72–0.78	0.75 ± 0.01					
SR	No stress	0.21–0.26	0.24 ± 0.01	**	*	NS	0.0001	0.66
	H	0.20–0.25	0.23 ± 0.01					
	H + D	0.20–0.25	0.23 ± 0.02					

*, **, NS indicate significance at 0.05 and 0.001 probability levels, and non-significant, respectively. SL, Seed length (mm); SW, Seed width (mm); SA, Seed area (mm^2^); SP, Seed perimeter (mm); SD, Seed diameter (mm); SE, Seed eccentricity (mm); SFD, Feret’s diameter; SC, Seed circularity; ST, Seed thickness; SR, Seed rugosity; H, heat, H + D, combined heat + drought; G, Genotype; T, Treatment.

**Table 5:** Mean value with standard deviation (Mean ± SD) of grain yield, seed ODAP content, and crude protein in three clusters under no stress, heat, and combined heat + drought conditions.

Treatment	Cluster	GYP	S-ODAP	CP
		Mean ± SD	Mean ± SD	Mean ± SD
No stress	Cluster I	3.28 ± 1.12	0.19 ± 0.05	22.51 ± 2.65
	Cluster II	2.46 ± 0.54	0.10 ± 0.02	20.96 ± 2.56
	Cluster III	4.42 ± 0.71	0.09 ± 0.02	25.48 ± 3.00
Heat stress	Cluster I	1.41 ± 0.16	0.14 ± 0.04	17.95 ± 1.94
	Cluster II	0.76 ± 0.11	0.13 ± 0.03	17.64 ± 2.62
	Cluster III	0.72 ± 0.13	0.27 ± 0.05	14.77 ± 3.50
Heat + Drought	Cluster I	0.87 ± 0.07	0.18 ± 0.04	14.59 ± 1.20
	Cluster II	0.42 ± 0.10	0.27 ± 0.04	13.56 ± 1.51
	Cluster III	0.56 ± 0.07	0.17 ± 0.04	15.17 ± 2.22

**Table 6:** Grasspea germplasm with stable performance in terms of grain yield, protein, and ODAP content.

Accession	Species	No Stress			Heat Stress			Heat + Drought Stress		
		GYP	S-ODAP	CP	GYP	S-ODAP	CP	GYP	S-ODAP	CP
66026	*L. tingitanus*	5.53	0.07	25.12	1.41	0.10	20.12	0.90	0.14	16.13
65018	*L. inconspicuus*	2.46	0.07	21.61	0.85	0.09	16.08	0.61	0.11	14.33
65687	*L. sativus*	2.79	0.07	21.39	1.54	0.10	16.52	0.84	0.13	14.00
118511	*L. sativus*	3.16	0.10	20.90	0.73	0.14	16.97	0.52	0.16	12.67
64931	*L. sativus*	4.37	0.11	27.62	0.68	0.11	23.37	0.54	0.16	14.98
65273	*L. annuus*	4.61	0.11	20.90	1.53	0.12	15.41	0.65	0.17	14.19

## Data Availability

Datasets generated and/or analyzed during the current study are available from the corresponding authors upon reasonable request.

## Supplementary Materials

The following supporting information can be downloaded at: https://www.mdpi.com/article/10.3390/plants12193501/s1, Table S1: Correlation between tested traits under no stress conditions, Table S2: Correlation between tested traits under heat stress conditions, Table S3: Correlation between tested traits under combined heat + drought stress conditions, Table S4: Percentage of contribution of different traits to the three major principal components with percentage variation under no stress, heat, and combined heat + drought stress, Table S5. Cluster mean ± SD of 20 evaluated traits under no stress (control), heat, and combined heat + drought stress conditions, Table S6. Description of the 24 accessions of 11 *Lathyrus* species used in the study.
