# Supplementary material for: Impact of Heat and Drought Stress on Grasspea and Its Wild Relatives
Source: Plants (Basel). 2023 Oct 8;12(19):3501. doi: 10.3390/plants12193501 (PMC10574926; doi:10.3390/plants12193501)
Supplement: Supplementary file 1 [file plants-12-03501-s001.zip › Plants-supplementary world tables.pdf]

Table S4. Percentage of contribution of different traits to the three major principal components with percentage variation under no stress, heat, and combined heat + drought stress.

Trait	**No stress (control)**			**Heat stress**			**Heat + Drought stress**		
	**PCA1**	**PCA2**	**PCA3**	**PCA1**	**PCA2**	**PCA3**	**PCA1**	**PCA2**	**PCA3**
Days to flowering	11.01	1.6	0.02	6.87	3.42	11.94	5.75	1.95	20.72
Days to first pod	8.26	3.08	0.36	5.49	0.81	18.38	5.6	3.07	17.85
Days to maturity	0.03	10.1	0.69	5.19	2.84	5.59	3.29	6.53	19.31
Leaf temperature	0.34	5.31	6.59	2.47	0.28	0.28	7.19	0.08	0.59
Relative leaf water content	0.58	1.89	5.13	9.21	0.38	0.57	9.29	0.21	0.79
Total chlorophyll	9.52	1.06	0.84	8.48	0.01	5.83	8.14	0.65	1
Proline content	2.68	6.76	0.02	10.03	2.06	1.24	6.79	0.03	0.42
Biological yield/plant	7.07	4.13	8.01	0.03	16.85	6.17	0.38	18.43	2.79
Plant height	5.26	9.11	5.01	1.65	7.39	2.43	0.19	9.68	0.59
Number of filled pods/plant	11.25	0.01	4.28	10.81	3.26	0.97	9.41	0	0.07
Number of unfilled pods/plant	0.48	13.3	8.22	5.82	3.42	1.19	5.09	0.5	0.11
Number of total pods/plant	6.15	0.7	7.58	11.04	3.38	0.03	8.58	0.54	1.89
Grain yield/plant	13.98	0.08	1.82	6.83	5.88	9.73	6.01	5.3	10.68
Number of seeds/plant	4.45	10.97	0.06	2.76	9.88	2.91	6.71	8.58	0.24
Number of seeds/pod	0.39	10.32	3.62	0.16	5.48	1.89	1.21	11.4	0.04
100-seed weight	9.12	4.57	3.71	3.36	14.64	5.25	0.06	20.33	10.55
Harvest index	4.19	11.18	3.34	1.31	10.1	8.43	7.5	3.16	4.52
Seed ODAP content	0.13	0.18	16.58	1.15	0.18	1.32	2.38	6.42	7.5
Leaf ODAP content	0.64	0.01	22.17	0.18	1.54	15.43	0.74	0.23	0.29
Crude protein content	4.46	5.63	1.93	7.16	8.19	0.42	5.68	2.93	0.06
Percentage variation (%)	28	18.98	13.71	32.36	15.6	11.01	42.15	16.38	8.82

Table S5. Cluster mean ± SD of 20 evaluated traits under no stress (control), heat, and combined heat + drought stress conditions.

	Cluster 1			Cluster 2			Cluster 3		
**Trait**	No stress	Heat	H+D	No stress	Heat	H+D	No stress	Heat	H+D
**DF**	55.67 ± 3.52	53.33 ± 7.02	45.57 ± 8.90	43.33 ± 2.88	40.10 ± 1.65	35.83 ± 4.94	44.68 ± 5.84	41.32 ± 3.84	37.17 ± 2.67
**DP**	63.23 ± 3.68	61.67 ± 5.51	51.87 ± 8.66	53.50 ± 2.15	48.71 ± 3.25	44.38 ± 3.82	52.54 ± 6.15	47.71 ± 3.98	43.54 ± 2.44
**DM**	84.87 ± 4.25	80.00 ± 4.36	73.07 ± 5.64	87.25 ± 3.88	73.62 ± 2.37	68.33 ± 1.94	83.51 ± 3.59	74.82 ± 3.18	67.91 ± 2.25
**LT**	27.45 ± 0.94	35.00 ± 1.16	38.27 ± 0.57	26.85 ± 0.81	34.09 ± 0.60	35.97 ± 1.56	27.45 ± 0.71	34.78 ± 1.37	36.05 ± 1.35
**RLWC**	81.38 ± 3.13	59.54 ± 5.05	49.05 ± 4.25	83.75 ± 2.25	72.26 ± 4.00	62.60 ± 3.07	79.23 ± 4.22	67.81 ± 3.83	61.13 ± 4.17
**Tchll**	15.25 ± 1.27	7.26 ± 0.90	4.29 ± 0.90	17.20 ± 0.39	12.68 ± 1.69	7.79 ± 2.68	15.98 ± 1.32	9.40 ± 2.03	7.20 ± 1.45
**PC**	4.11 ± 1.42	2.92 ± 0.15	2.65 ± 1.19	6.52 ± 1.68	20.15 ± 4.44	16.14 ± 5.20	3.84 ± 1.35	13.32 ± 3.48	14.25 ± 5.85
**BYP**	6.17 ± 2.83	4.77 ± 3.01	2.43 ± 0.58	10.82 ± 1.10	5.02 ± 1.01	2.33 ± 0.14	6.62 ± 1.50	3.17 ± 0.82	2.91 ± 0.75
**PLH**	41.13 ± 9.22	28.33 ± 7.84	33.27 ± 6.17	74.08 ± 21.98	38.68 ± 4.65	27.63 ± 5.64	47.75 ± 10.26	36.89 ± 7.94	32.30 ± 4.87
**NFPP**	9.47 ± 2.03	4.83 ± 1.61	3.20 ± 0.84	11.94 ± 0.68	6.61 ± 0.58	4.67 ± 0.42	11.38 ± 1.91	6.04 ± 0.44	4.66 ± 0.39
**NUPP**	5.00 ± 1.01	4.94 ± 0.86	4.72 ± 1.02	5.24 ± 0.98	5.70 ± 0.33	5.82 ± 0.90	4.61 ± 0.39	5.63 ± 0.57	5.50 ± 0.21
**NTPP**	14.47 ± 2.93	10.11 ± 1.90	7.61 ± 1.96	16.24 ± 2.72	12.31 ± 0.61	10.49 ± 0.87	16.06 ± 1.85	11.67 ± 0.85	10.06 ± 0.62
**GYP**	2.15 ± 0.55	0.73 ± 0.07	0.35 ± 0.07	4.10 ± 1.05	1.44 ± 0.13	0.56 ± 0.09	3.17 ± 0.98	0.79 ± 0.16	0.65 ± 0.19
**SN**	24.73 ± 5.40	10.33 ± 2.08	4.62 ± 2.08	22.94 ± 3.84	11.58 ± 2.48	10.12 ± 1.96	28.74 ± 7.01	10.30 ± 2.11	6.54 ± 1.30
**SNPP**	2.73 ± 0.91	2.00 ± 0.00	1.46 ± 0.51	1.95 ± 0.41	1.75 ± 0.38	2.16 ± 0.28	2.49 ± 0.39	1.71 ± 0.30	1.41 ± 0.27
**HSW**	8.73 ± 1.14	6.71 ± 1.25	8.31 ± 1.89	16.50 ± 2.64	13.17 ± 4.31	5.55 ± 0.57	10.91 ± 1.61	7.78 ± 1.42	10.21 ± 3.28
**HI**	36.84 ± 6.37	27.78 ± 18.39	15.57 ± 5.25	38.57 ± 9.22	30.15 ± 4.95	26.67 ± 3.53	47.54 ± 8.37	25.44 ± 3.16	22.88 ± 4.35
**S-ODAP**	0.16 ± 0.09	0.18 ± 0.06	0.28 ± 0.04	0.14 ± 0.03	0.14 ± 0.04	0.28 ± 0.06	0.11 ± 0.04	0.17 ± 0.07	0.18 ± 0.04
**L-ODAP**	0.27 ± 0.12	0.25 ± 0.01	0.40 ± 0.14	0.24 ± 0.03	0.30 ± 0.07	0.58 ± 0.16	0.26 ± 0.07	0.39 ± 0.12	0.41 ± 0.12
**CP**	20.36 ± 2.92	13.68 ± 3.59	12.63 ± 1.64	20.88 ± 0.94	18.03 ± 2.08	15.68 ± 2.75	23.41 ± 3.17	17.65 ± 2.36	14.47 ± 1.07

DF, days to flowering; DP, days to first pod; DM, days to maturity; BYP, biological yield plant^{-1}; LT, leaf temperature; RLWC, relative leaf water content; Tchll, total chlorophyll; PC, proline content; PLH, plant height; NFPP, number of filled pods plant^{-1}; NUPP, number of unfilled pods plant^{-1}; NTPP, number of total pods plant^{-1}; GYP, grain yield plant^{-1}; SN, seed number plant^{-1}; SNPP, seed number pod^{-1}; HSW, 100-seed weight; HI, harvest index; S-ODAP, seed ODAP content; L-ODAP, leaf ODAP content; CP, crude protein.; H, individual heat, H+D, combined heat-drought.

Table S6. Description of the 24 accessions of 11 *Lathyrus* species used in the study

Accession	*Species*	Origin	DOI	Latitude	Longitude
IG 64892	*L. sativus*	Greece	10.18730/7GCF=	37.47	22.33
IG 64931	*L. sativus*	Iran	10.18730/7GDP0	35.17	48.55
IG 64954	*L. sativus*	Afghanistan	10.18730/7GEBN	34.82	67.52
IG 65018	*L. 3icero3icuous*	Turkey	10.18730/7GG33	37.85	41.17
IG 65117	*L. sativus*	Ethiopia	10.18730/7GJA0	Not available	Not available
IG 65133	*L. sativus*	Ethiopia	10.18730/7GJTG	Not available	Not available
IG 65273	*L. annuus*	Syria	10.18730/7GQ57	34.58	36.73
IG 65369	*L. blepharicarpus*	Syria	10.18730/7GT0R	35.60	36.05
IG 65671	*L. sativus*	Pakistan	10.18730/7H24S	27.57	68.22
IG 65673	*L. sativus*	Pakistan	10.18730/7H26V	28.07	69.73
IG 65687	*L. sativus*	Turkey	10.18730/7H2J2	39.92	32.83
IG 66026	*L. tingitanus*	Australia	10.18730/7HBCS	Not available	Not available
IG 66049	*L. ciliolatus*	Jordan	10.18730/7HC19	30.68	35.62
IG 66054	*L. hierosolymitanus*	Turkey	10.18730/7HC5D	36.83	36.82
IG 66065	*L. gorgoni*	Jordan	10.18730/7HCDN	32.62	35.85
IG 110632	*L. sativus*	Syria	10.18730/8R285	37.01	41.68
IG 114526	*L sativus*	Bangladesh	10.18730/8VM9W	23.43	89.88
IG 114531	*L. sativus*	Bangladesh	10.18730/8VME~	23.46	89.93
IG 114595	*L. sativus*	Bangladesh	10.18730/8VPEQ	24.95	89.93
IG 114990	*L. aphaca*	Nepal	10.18730/8W18U	26.46	87.55
IG 118511	*L. sativus*	Ethiopia	10.18730/8Z54D	10.01	38.25
Jabboulah	*L. 3icero*	Lebanon	Released variety	Not available	Not available
L.OCH	*L. ochrus*	Not available	Not available	Not available	Not available
LAT 495	*L. sativus*	Slovakia	10.25642/IPK/GBIS/55042	Not available	Not available
